# The prevalence of women’s emotional and physical health problems following a postpartum haemorrhage: a systematic review

**DOI:** 10.1186/s12884-016-1054-1

**Published:** 2016-09-05

**Authors:** Margaret Carroll, Deirdre Daly, Cecily M. Begley

**Affiliations:** 1 School of Nursing and Midwifery, Trinity College Dublin, 24 D’Olier Street, Dublin, DO2 T283 Ireland; 2 Institute of Health and Care Sciences, Sahlgrenska Academy, University of Gothenburg, Gothenburg, Sweden

**Keywords:** Postpartum haemorrhage, Systematic review, Emotional health problems, Physical health problems

## Abstract

**Background:**

Postpartum Haemorrhage (PPH) is a leading cause of maternal mortality with approximately 225 women dying as a result of it each day especially in low income countries. However, much less is known about morbidity after a PPH. This systematic review aimed to determine the overall prevalence of emotional and physical health problems experienced by women following a postpartum haemorrhage.

**Methods:**

Eight databases were searched for published non-randomised, observational, including cohort, primary research studies that reported on the prevalence of emotional and/or physical health problems following a PPH. Intervention studies were included and data, if available, were abstracted on the control group. All authors independently screened the papers for inclusion. Of the 2210 papers retrieved, six met the inclusion criteria. Data were extracted independently by two authors. The methodological quality of the included studies was assessed using a modified Newcastle Ottawa Scale (NOS). The primary outcome measure reported was emotional and physical health problems up to 12 months postpartum following a postpartum haemorrhage.

**Results:**

Two thousand two hundred ten citations were identified and screened with 2089 excluded by title and abstract. Following full-text review of 121 papers, 115 were excluded. The remaining 6 studies were included. All included studies were judged as having strong or moderate methodological quality. Five studies had the sequelae of PPH as their primary focus, and one study focused on morbidity postnatally, from which we could extract data on PPH. Persistent morbidities following PPH (at ≥ 3 and < 6 months postpartum) included postnatal depression (13 %), post-traumatic stress disorder (3 %), and health status ‘much worse than one year ago’ (6 %). Due to the different types of health outcomes reported in the individual studies, it was possible to pool results from only four studies, and only then by accepting the slightly differing definitions of PPH. Those that could be pooled reported rates of acute renal failure (0.33 %), coagulopathy (1.74 %) and re-admission to hospital following a PPH between 1 and 3 months postpartum (3.6 %), an appreciable indication of underlying physical problems.

**Conclusion:**

This systematic review demonstrates that the existence and type of physical and emotional health problems post PPH, regardless of the volume of blood lost, are largely unknown. Further large cohort or case control studies are necessary to obtain better knowledge of the sequelae of this debilitating morbidity.

**Electronic supplementary material:**

The online version of this article (doi:10.1186/s12884-016-1054-1) contains supplementary material, which is available to authorized users.

## Background

Postpartum Haemorrhage (PPH) is recognised as one of the leading causes of maternal mortality worldwide, accounting for 1 in 4 maternal deaths globally, but much less is known about morbidity post PPH. At least 225 women die from PPH every day, the majority of these deaths occurring in low income countries [1]. In the most recent Confidential Enquiries into Maternal Deaths (2011–13) in the UK, PPH was identified as being responsible for 10 % of direct maternal deaths [2]. Historically, maternal mortality has been used as an indicator of the quality of maternity services; however, as women’s general health improves, and preventative care and treatment for PPH become more accessible, more women are surviving PPH, making the evaluation of morbidity a useful complementary measure.

Although PPH is itself a maternal morbidity, the outcomes for women who experience a PPH have not been studied widely. A surveillance study conducted in 27 obstetric units in Brazil found the maternal near miss ratio to be 2.2 per 1000 women with PPH [3]. Wagner et el. [4] describe a review of 11 studies, showing that PPH resulted in anaemia with a summary ratio of 2.35 (95 % CI 1.44–3.84), albeit with considerable heterogeneity. Emotional outcomes occur also, with the majority of women who experience PPH having negative memories of the birth including, for some, a persistent fear of dying [5]. A systematic review of women’s experiences of all types of severe maternal morbidity showed that women found these incidents physically and emotionally distressing [6], and therefore worthy of study.

The prevalence of PPH varies, with the worldwide prevalence thought to be between 6 and 11 % [7]. In England, the incidence increased from 7 % in 2004/5 to 13 % in 2011/12 [8]. In an eleven-year population-based cohort study in Ireland, the rate of PPH increased from 1.5 % in 1999 to 4.1 % in 2009 [9]. In Australia (NSW), the PPH rate increased from 6.1 % in 2003 to 8.3 % in 2011 [10].

More recently, the rate in one large Dublin maternity hospital was 14.6 % in 2014, a 4-fold increase from the 3.3 % incidence reported in 2008 [11]. Whilst some of these increases may be a result of more accurate estimation of blood loss, or because PPH was redefined with a lower threshold of blood loss, it never-the-less means that more women are experiencing PPH.

Given the prevalence of PPH and approximately 135 million births occurring worldwide each year, approximately 8–11 million women will experience a PPH and many will suffer some health sequelae. The focus of this systematic review, therefore, was on determining the overall prevalence of emotional and physical health problems experienced by women following a postpartum haemorrhage.

### Defining postpartum haemorrhage

PPH is defined in four different ways by WHO [1], RCOG [12], ICD-10 codes [13] and NICE [14]. Primary (sometimes referred to as immediate or early) occurs within 24 h of birth, whilst secondary (sometimes referred to as delayed or late) occurs after 24 h and up to 12 weeks postpartum.

In these four definitions, the common component is the inclusion of blood loss greater than 500 mls for a primary PPH. For secondary PPH, only the ICD-10 codes [13] categorise blood loss as being greater than 500 mls. The terms used in two other definitions state ‘*abnormal or excessive bleeding from the birth canal*’ [12] / or ‘*of the amount that adversely affects the maternal physiology*’ [13]. WHO [1] or NICE [14] do not define secondary PPH.

Other terms used to define PPH include major obstetric haemorrhage, defined not only according to blood loss (>2500 mls), but also by number of transfused units of blood (*n* = 5) or requiring coagulopathy treatment (e.g., fresh frozen plasma, fibrinogen concentrate substitution therapy, platelets) [15, 16].

However, despite the definition used, being able to estimate blood loss accurately is recognised as being almost clinically impossible [17], and studies have shown that blood loss is overestimated at low volumes and underestimated when the volume is large thus underestimating the severity of the PPH [18–20].

### Defining maternal morbidity - women’s physical and emotional health problems

The term ‘maternal morbidity’ encompasses a wide range of acute and chronic conditions, ranging from ‘near death’ or ‘near miss’ to non-life threatening events. A WHO review, undertaken to create a consensus around a definition of maternal morbidity, found significant discrepancies in the literature and amongst experts [21]. More recently, population based studies have reported prevalence of severe maternal morbidity and, according to these reports, 1 in 140 pregnant women will suffer from a severe morbidity, the most common being PPH [16]. In Ireland, major obstetric haemorrhage was also the most common cause of severe maternal morbidity, with a rate of 2.55 per 1000 maternities [15]. These rates are for severe haemorrhage only, meaning that the prevalence of any degree of PPH in the wider maternity population is much greater.

## Methods

### Aim and objectives

This systematic review aimed to determine the overall prevalence of emotional and physical health problems experienced by women following a postpartum haemorrhage. The objectives were:

To determine the prevalence of women’s emotional health problems following a postpartum haemorrhage up to 3 months, >3 months and ≤6 months, and >6 months and ≤12 months postpartum; and

To determine the prevalence of women’s physical health problems following a postpartum haemorrhage up to 3 months, >3 months and ≤6 months, and >6 months and ≤12 months postpartum.

#### Outcomes

Primary outcome measures were the reported emotional and physical health problems up to 12 months postpartum following a postpartum haemorrhage. The type of haemorrhage experienced included: 1. Primary / immediate PPH; 2. Secondary / delayed PPH; and 3. PPH according to definition (as defined by WHO [1], RCOG [12], ICD-10 codes [13] and NICE [14]). When studies did not cite any of the above references, their definition was abstracted and assessed.

Secondary outcome measures were women’s physical and/or emotional health problems following PPH according to parity, age, BMI and mode of birth and amount of blood lost/severity of PPH.

#### Inclusion and exclusion criteria

Inclusion and exclusion criteria were determined a priori*.* Eligible participants were postpartum women, irrespective of parity, who had given birth to a baby of at least 24 weeks gestation and who experienced a postpartum haemorrhage. An individual study had to report primary data on prevalence of emotional and/or physical health problems at any time point up to 12 months postpartum, to be included.

Published non-randomised, observational including cohort primary research studies, reporting on prevalence of emotional and/or physical health problems following a PPH were included. Intervention studies were included and data, if available, were abstracted on the control group.

Case reports, reviews and qualitative studies were excluded.

#### Search and selection strategy

A computerised search of the following electronic databases was performed from their foundation to April 2015: CINAHL , PubMED (MEDLINE not searched as included in PubMED), Maternity and Infant Care, PsycINFO, EMBASE (also includes deduplicated MEDLINE citations from 1966 to the present), Web of Science, Social Science Index and The Cochrane Library. Key words, search terms and string were developed, tested and adapted for each database. The search was not restricted by year or English language publications. Search terms were developed for the three key terms: ‘postpartum haemorrhage’, ‘postnatal period’ and ‘women’s emotional and physical health’ (Table 1). Search strings for individual databases were created (Additional file 1: Search Strings for Databases).

**Table 1:** Key words and search terms and string

Key words	Search terms/String
Postnatal period	TI postnatal OR AB postnatal OR TI post natal OR AB post natal OR TI post-natal OR AB post-natal OR TI postpartum OR AB postpartum OR TI post partum OR AB post partum OR TI post-partum OR AB post-partum OR TI “postnatal period” OR AB “postnatal period” OR TI “post natal period” OR AB “post natal period” OR TI “post-natal period” OR AB “post-natal period” OR TI “postpartum period” OR AB “postpartum period” OR TI “post partum period” OR AB “post partum period” OR TI “post-partum period” OR AB “post-partum period” OR TI puerperium OR AB Puerperium OR TI peripartum OR AB peripartum OR TI peri partum OR AB peri partum OR TI peri-partum OR AB peri-partum OR TI “peripartum period” OR AB “peripartum period” OR TI “peri partum period” OR AB “peri partum period” OR TI “peri-partum period” OR AB “peri-partum period” OR TI peripartum period OR AB peripartum period
Postpartum haemorrhage	TI PPH OR AB PPH OR TI postpartum haemorrhage OR AB postpartum haemorrhage OR TI post partum haemorrhage OR AB post partum haemorrhage OR TI post-partum haemorrhage OR AB post-partum haemorrhage OR TI postpartum hemorrhage OR AB postpartum hemorrhage OR TI post partum hemorrhage OR AB post partum hemorrhage OR TI post-partum hemorrhage OR AB post-partum hemorrhage OR TI obstetric haemorrhage OR AB obstetric haemorrhage OR TI obstetric hemorrhage OR AB obstetric hemorrhage OR TI major obstetric haemorrhage OR AB major obstetric haemorrhage OR TI major obstetric hemorrhage OR AB major obstetric hemorrhage OR TI massive haemorrhage OR AB massive haemorrhage OR TI massive hemorrhage OR AB massive hemorrhage OR TI massive obstetric haemorrhage OR AB massive obstetric haemorrhage OR TI massive obstetric hemorrhage OR AB massive obstetric hemorrhage OR TI severe haemorrhage OR AB severe haemorrhage OR TI severe hemorrhage OR AB severe hemorrhage OR TI severe obstetric haemorrhage OR AB severe obstetric haemorrhage OR TI severe obstetric hemorrhage OR AB severe obstetric hemorrhage OR TI severe blood loss OR AB severe blood loss
Women’s emotional and physical health problems	TI women’s health OR AB women’s health OR TI womens health OR AB womens health OR TI woman’s health OR AB woman’s health OR TI womans health OR AB womans health OR TI health outcome OR AB health outcome OR TI health outcomes OR AB health outcomes OR TI adverse outcome OR AB adverse outcome OR TI adverse outcomes OR AB adverse outcomes OR TI pregnancy outcome OR AB pregnancy outcome OR TI pregnancy outcomes OR AB pregnancy outcomes OR TI puerperal disorder OR AB puerperal disorder OR TI puerperal disorders OR AB puerperal disorders OR TI maternal morbidity OR AB maternal morbidity OR TI maternal morbidities OR AB maternal morbidities OR
	TI mental health OR AB mental health OR TI mental disorder OR AB mental disorder OR TI mental disorders OR AB mental disorders OR TI mental disease OR AB mental disease OR TI mental diseases OR AB mental diseases OR TI emotional wellbeing OR AB emotional wellbeing OR TI emotional well being OR AB emotional well being OR TI emotional well-being OR AB emotional well-being OR TI emotional health OR AB emotional health OR TI postnatal depression OR AB postnatal depression OR TI post natal depression OR AB post natal depression OR TI post-natal depression OR AB post-natal depression OR TI postpartum depression OR AB postpartum depression OR TI post partum depression OR AB post partum depression OR TI post-partum depression OR AB post-partum depression OR TI postnatal distress OR AB postnatal distress OR TI post natal distress OR AB post natal distress OR TI post-natal distress OR AB post-natal distress OR TI postpartum distress OR AB postpartum distress OR TI post partum distress OR AB post partum distress OR TI post-partum distress OR AB post-partum distress OR TI postnatal stress OR AB postnatal stress OR TI post natal stress OR AB post natal stress OR TI post-natal stress OR AB post-natal stress OR TI perinatal mental health OR AB perinatal mental health OR TI perinatal mental health problems OR AB perinatal mental health problems OR TI peri natal mental health problems OR AB peri natal mental health problems Or TI perinatal mental health problem OR AB perinatal mental health problem OR TI peri natal mental health problem OR AB peri natal mental health problem OR TI postnatal anxiety OR AB postnatal anxiety OR TI post natal anxiety OR AB post natal anxiety OR TI post-natal anxiety OR AB post-natal anxiety OR TI postpartum anxiety OR AB postpartum anxiety OR TI post partum anxiety OR AB post partum anxiety OR TI post-partum anxiety OR AB post-partum anxiety OR OR TI postnatal anxieties OR AB postnatal anxieties OR TI post natal anxieties OR AB post natal anxieties OR TI post-natal anxieties OR AB post-natal anxieties OR TI postpartum anxieties OR AB postpartum anxieties OR TI post partum anxieties OR AB post partum anxieties OR TI post-partum anxieties OR AB post-partum anxieties OR TI postnatal psychosis OR AB postnatal psychosis OR TI post natal psychosis OR AB post natal psychosis OR TI post-natal psychosis OR AB post-natal psychosis OR TI postpartum psychosis OR AB postpartum psychosis OR TI post partum psychosis OR AB post partum psychosis OR TI post-partum psychosis OR AB post-partum psychosis OR
	TI physical health OR AB physical health OR TI physical health problem OR AB physical health problem OR TI physical health problems OR AB physical health problems OR TI health problem OR AB health problem OR TI health problems OR AB health problems

Following an initial scoping search, the search was limited to ‘title’ OR ‘abstract’. The appropriate search terms were combined with the Boolean operands ‘AND’ and ‘OR’ as appropriate.

Reference lists of papers retrieved for full text review were also reviewed for potentially eligible papers that may not have been captured by the electronic search strategy [22].

#### Data management processes

The review followed the processes recommended by Higgins and Green [23]. Following agreement on the final search string, the selection of eligible studies and data extraction were performed by three people independently (MC, CB and DD), and the final results compared, thus minimising the likelihood that errors would go undetected. MC, CB and DD have expertise in midwifery, CB has systematic review and statistical expertise, DD has systematic review expertise.

#### Quality assessment of included studies

The Newcastle Ottawa Scale (NOS) [24] was modified and a scale of 0–6 stars was used to assess methodological quality of included studies (Table 2). Six stars was deemed as strong, four to five stars was deemed as moderate and three or fewer stars was deemed as weak methodological quality. Studies judged to have weak methodological quality were excluded from the data extraction process. All items were regarded as equally important, and no weighting was applied to any item or category.

**Table 2:** Quality assessment tool – Modified Newcastle Ottawa Scale (NOS)

Modified Newcastle Ottawa Scale (NOS)	Quality Assessment Tool – Modified Newcastle Ottawa Scale (NOS)								
	1	2	3	4	5	6	7	8	9
	Representativeness of the exposed cohort (postpartum women)	Do the authors provide a definition of PPH?	Ascertainment of exposure	Assessment of outcome	Was the follow-up long enough for outcome to occur? (From birth up to 12 months postpartum or postnatal period defined)	Adequacy of follow-up	If necessary and/or appropriate provide details explaining decision on adequacy of follow-up	How many stars (^a^) does this paper receive?	Based on the overall quality, do you wish to include this paper?
	Truly representative^a^	Yes^a^	Secure record^a^	Independent blind assessment^a^	Yes^a^	Complete follow-up^a^			✓ = Yes, include
	Somewhat representative^a^	No	Record linkage/Questionnaire ^a^	Record linkage/Questionnaire^a^	No	Subjects lost to follow-up <20 %^a^			No = Exclude
	Selected group of users		Descriptive written self-report	Descriptive self-report		Subjects lost to follow-up >20 %			An overall rating of 3 or less implies weak quality and should be excluded
	No clear description		No description	No description		No statement			
						Not applicable^a^			

^a^One star awarded

#### Data extraction management

The following data were abstracted and inserted in a data collection form, designed a priori.

##### Primary outcome measures

i. Country of study, study design, date of data collection, and methods used
ii. Definition of PPH, if any, used.

The number of women reported as having a PPH, and who also reported a particular physical and/or emotional health problem, and the postpartum time it was reported.

### Data analysis

Where possible, data reported on similar outcomes at the same time points were pooled.

## Results

### Results of search and selection strategy

**Fig. 1:**
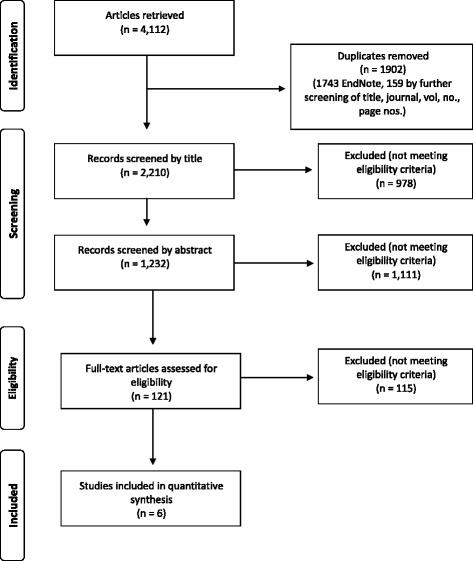
Flow diagram of the search and selection process

Following removal of duplicates, a total of 2210 papers were retrieved (Fig. 1), and 978 were excluded on title screening. A further 1111 were excluded on abstract screening, and the remaining 121 papers were subjected to full text review. Citations not in English were screened at this stage using Google translate. A total of 12 papers appeared to meet the inclusion criteria for this review [25–36]; however, at data extraction stage it was realised that we were unable to abstract data from six papers [26, 29, 32–35] because the outcomes reported in these papers all related to management or treatments of PPH, and no maternal physical or emotional health outcomes were reported.

### Characteristics of included studies

The characteristics of the included studies are illustrated in Table 3. Five studies had the sequelae of PPH as their primary focus. The remaining study [27] focused on morbidity postnatally and we were able to extract data on the population of women who experienced a PPH. The majority of studies took place between 1995 and 2007, with one study conducted in 1989 (Table 3). Four studies collected data over 1 to 3 years, and two studies over a 5–9 year time-frame (Table 3).

**Table 3:** Characteristics and quality assessment of included studies

Reference	Country	OECD country (at the time research was conducted)	Year of study	Study design	ICD-10 (2010) or similar definition of PPH used	Timing/severity/amount of PPH used in definition	Number and description of participants included	Data collection	Data reported and timing (HP, <1, ≥1 < 3, ≥3 < 6, ≥6 < 12, =12 months)	Quality assessment
Chauleur et al. (2008) [25]	France	Yes	1999–2004	P, C	Similar	Timing and severity not amount of bleeding	*n* = 32,463, Primigravida women (women in their first intended pregnancy)	D, MR	HP at <1, ≥1 < 3	Strong
Thompson et al. (2011) [27]	Australia and New Zealand	Yes	2006–07	P, C	Similar	√	*n* = 206, Primiparous and Multiparous women who returned the 1st survey	MR, Q	HP at ≥1 < 3, ≥3 < 6	Strong
Bateman et al. (2010) [28]	United States	Yes	1995–2004	R, C	ICD-9	√	*n* = 25,654, Primiparous and Multiparous women who had a PPH	MR (national data)	HP at <1	Moderate
Liu et al. (2002) [30]	Canada	Yes	1989–99	R, C	ICD-9	√	*n* = 2,652,726, Primiparous and Multiparous women who gave birth during a 11 year period	MR (national data)	HP at ≥1 < 3	Strong
Naz et al. (2008) [31]	Pakistan	No	2006	P, C	Not stated	Severity of bleeding but not amount or timing	*n* = 705, Primiparous and Multiparous women who had a Primary PPH	MR	HP at <1	Moderate
Hoveyda and MacKenzie (2001) [36]	England	Yes	1996–98	R	Similar	Severity and timing of bleeding but not amount	*n* = 19,136, Primiparous and Multiparous women with Primary PPH, and those with Secondary PPH	MR	HP at <1	Strong

Explanation: *P* prospective, *C* cohort, *R* retrospective, *MR* medical records, *Q* questionnaire, *D* diary, *HP* health problems

### Results of methodological quality assessment

All included studies were judged as having strong or moderate methodological quality (Table 3). The two studies judged as having only moderate methodological quality were deemed so because PPH and/or the postnatal period were not defined in the publication.

### Results of data extraction

The six included papers reported data from six individual studies conducted in seven countries. Five studies were conducted in OECD countries and one in a non-OECD country. Detailed characteristics of included papers are presented in Table 3. Five studies reported the outcomes of primary PPH [25, 27, 28, 30, 31] and one reported outcomes after secondary PPH [36].

### Definitions of PPH

Studies varied in how they defined PPH, and these were compared with the definitions provided by WHO [1], RCOG [12], ICD-10 codes [13] and NICE [14]. One study, which was reporting outcomes after severe or major haemorrhage, stating it as ≥1,500mls, defined PPH in the traditional manner, by amount of blood lost [27]. Two studies [28, 30] used the ICD-9 codes to define PPH which also includes *“the amount that adversely affects the maternal physiology, such as blood pressure and haematocrit”*, although these parameters are not defined. Three studies defined PPH in a somewhat similar fashion, including treatment required, e.g., *“bleeding … persisting after manual exploration of the uterine cavity and requiring I.V. prostaglandin administration”* [25], *“findings of pelvic examination, condition of the uterus”* [31], *“a fall in haemoglobin, for example, to 7 g/dl and/or by 4 g/dl or more”* [25, 27].

### Outcomes of PPH

One study reported on outcomes post secondary PPH, providing a definition similar to the RCOG’s (2014) [36]. In total, 44 outcomes were reported in the six included papers (see Tables 4, 5 and 6 for a list and timeline of the health outcomes). The number of reported health problems varied from one (the rate of re-admission at ≥1 but <3 months postpartum [30]), to 31 [27]. Four studies reported health outcomes in the 1st postpartum month [25, 28, 31, 36], two at ≥1 < 3 [27, 30], and one at ≥3 < 6 [27]. Five reported outcomes following primary PPH [25, 27, 28, 30, 31] and one following both primary and secondary PPH [36].

**Table 4:** Reported health problems up to one month postpartum

References	Chauleur et al. (2008) [25]	Bateman et al. (2010) [28]	Naz et al. (2008) [31]	Hoveyda & MacKenzie (2001) [36]	Hoveyda & MacKenzie (2001) [36]
Number of participants	*n* = 317	*n* = 25,654	*n* = 50	*n* = 657 (primary PPH)	*n* = 132 (secondary PPH)
Reported Health Problems up to 1 month postpartum					
Acute Renal Failure		82 (0.32 %)	2 (4 %)		
Acute Respiratory Failure		105 (0.41 %)			
Anaemia			20 (40 %)		
Anaemia + DIC			5 (10 %)		
Anaemia + DIC + ARF			1 (2 %)		
Coagulopathy / DIC		445 (1.73 %)	1 (2 %)		
Length of stay more than 7 days		656 (2.56 %)			
Perforation of the uterus					3 (2.27 %)
Prolonged mechanical ventilation (>96 h)		13 (0.05 %)			
Secondary PPH				33 (5.02 %)	
Sepsis / Infection		25 (0.1 %)			
Shock + Anaemia			2 (4 %)		
Superficial venous thrombosis	3 (0.9 %)^a^				

^a^Primiparous women only

**Table 5:** Reported health problems ≥ 1 month and < 3 months postpartum

References	Thompson et al. (2011) [27]	Liu et al. (2002) [30]
Number of participants	*n* = 171	*n* = 113,861
Reported Health problems ≥ 1 < 3 months postpartum		
Anxiety: State Trait Anxiety Inventory (median)	10	
Depression: Edinburgh Postnatal Depression Scale > 12	19 (11 %)	
Post-traumatic stress disorder: PCL > 44	9 (5 %)	
Health Status: SF36 ‘somewhat worse than 1 year ago’ ago’	46 (26.9 %)	
Health Status: SF36 ‘much worse than 1 year ago’	14 (8.2 %)	
Physical problems:		
i. Painful perineum (vaginal births only)	54/103 (52.5 %)	
ii. Perineal infection (vaginal births only)	6/104 (5.8 %)	
iii. Pain at CS site (CS births only)	53/65 (81.5 %)	
iv. CS wound infection (CS births only)	12/65 (18.5 %)	
v. Uterine infection	10 (5.9 %)	
vi. Urinary tract infection	11 (6.5 %)	
vii. Stress urinary incontinence	57 (33.5 %)	
viii. Constipation	81 (47.6 %)	
ix. Incontinence of faeces	10 (5.9 %)	
x. Incontinence of flatus	31 (18.5 %)	
xi. Breast infection/ mastitis	37 (21.8 %)	
xii. Backache	92 (54.7 %)	
xiii. Headache (frequent)	49 (29.2 %)	
xiv. Physical exhaustion	119 (71.2 %)	
Re-admission Reasons:	17 (10 %)	4108 (3.6 %)
i. Mastitis/Breast abscess	4/17	
ii. Bleeding/Retained of products of conception	3/17	
iii. Low haemoglobin/transfusion	3/17	
iv. Problems with perineal stitches	2/17	
v. Postnatal depression, hyperventilation, pleuritic pain, blood clot on liver, severe migraine, neonatal jaundice	1/17 (each)	

*CS* caesarean section

**Table 6:** Reported health problems ≥ 3 month and < 6 months postpartum

References	Thompson et al. (2011) [27]
Number of participants	*n* = 171
Anxiety: State Trait Anxiety Inventory (median)	10
Depression: Edinburgh Postnatal Depression Scale > 12	21 (13 %)
Post-traumatic stress disorder: PCL > 44	5 (3 %)
Health Status: SF36 ‘somewhat worse than 1 year ago’	51 (30.7 %)
Health Status: SF36 ‘much worse than 1 year ago’	10 (6 %)
Physical problems:	
i. Painful perineum (vaginal births only)	25/100 (25 %)
ii. Perineal infection (vaginal births only)	0
iii. Pain at CS site (CS births only)	29/60 (48.3 %)
iv. CS wound infection (CS births only)	5/61 (8.2 %)
v. Uterine infection	4 (2.4 %)
vi. Urinary tract infection	6 (3.7 %)
vii. Stress urinary incontinence	42 (25.6 %)
viii. Constipation	39 (24 %)
ix. Incontinence of faeces	5 (3 %)
x. Incontinence of flatus	26 (16.1 %)
xi. Breast infection/ mastitis	22 (13.6 %)
xii. Backache	99 (60 %)
xiii. Headache (frequent)	51 (30.9 %)
xiv. Physical exhaustion	87 (52.7 %)
Re-admission Reasons:	4 (2 %)
i. Bleeding/ Retained of products of conception	2/4
ii. Post-natal depression	1/4
iii. Cholecystitis	1/4

*CS* caesarean section

The health outcomes reported in the early postpartum period tended to be acute, i.e., renal failure, respiratory failure, prolonged ventilation, coagulopathy/Disseminated Intravascular Coagulation (DIC), sepsis, anaemia, Superficial Venous Thrombosis and perforation of the uterus. Health problems reported in later months tended to be less severe, and some had become chronic.

Thompson et al. [27] was the only study that reported the prevalence of both emotional and physical health problems following a significant (>1,500mls) primary PPH between one and six months postpartum (Tables 5 and 6). The emotional health problems reported were anxiety, postnatal depression, fatigue, post-traumatic stress disorder and general health status, measured using Speilberger State-Trait Anxiety Inventory, Edinburgh Postnatal Depression Scale, Milligan’s 10-item Scale for fatigue, 17-item PTSD Checklist and 360 item short form General Health Survey. The physical problems reported were infection (perineal, caesarean section (CS) wound, uterine, breast), pain (perineal, site of CS scar), urinary tract infection, incontinence (stress urinary, faeces, flatus), constipation, backache, headaches (frequent) and physical exhaustion. Women rated these as ‘not a problem’, ‘a minor problem’, or ‘a major problem’.

Only two studies compared women who had primary PPH with those who did not. Chauleur et al. [25] found that women who experienced PPH were at increased risk of superficial venous thrombosis (adjusted relative risk: 5.3, 95 % CI 1.6 to 17). Hoveyda & MacKenzie [36] analysed data from 657 women with primary PPH and found 33 (5.02 %) had a secondary PPH compared with only 99 out of 18,479 women who did not have primary PPH (0.54 %), giving an odds ratio of 9.3 (95 % CI 6–2 to 14). One other study [27] provided data on those women re-admitted to hospital following a PPH at ≥ 1 month and < 3 months postpartum (4108 out of 113,861, 3.61 %). We compared these results with their data on the cohort of women who did not have PPH and were re-admitted (30,229 out of 2,538,865, 1.19 %). The difference was statistically significant with an odds ratio of 3.1 (95 % CI 3.01 to 3.21), *p* < 0.0001.

Data on sub-groups of women, grouped by parity, age and BMI, following postpartum haemorrhage were not reported in any paper. Due to the different types of health outcomes reported in the individual studies, it was possible to pool results from only four studies, and only then by accepting the slightly differing definitions of PPH. Combining the results of Bateman et al. [28] and Naz et al. [31] gave a total of 25,704 women. Acute renal failure occurred in 84 women (0.33 %) and coagulopathy in 446 (1.74 %). However, it should be noted that the study by Naz et al. [31] was conducted on 50 women who had primary PPH, of whom 10 (20 %) had a subtotal hysterectomy performed, a population rate of peripartum hysterectomy (1.42 %) far in excess of the prevalence found in other countries. It is likely that the high incidence of acute renal failure and other complications seen in this study are due to the surgical procedure, rather than PPH *per se*. The benefit of combining these results is that a more realistic overall prevalence is reached.

The combined results of Liu et al. [30] and Thompson et al. [27] in relation to those re-admitted to hospital following a PPH at ≥ 1 month and < 3 months postpartum, gave a total of 4125 re-admissions out of 114,032 (3.6 %).

## Discussion

There are few papers that report on health outcomes following a postpartum haemorrhage, despite the reported increase in the incidence of PPH and more severe PPH, across the globe [8–10]. Many papers focus only on the risks for, and prevention of, maternal mortality due to PPH, with scant regard for surviving women’s physical and emotional state. Women’s health and level of possible morbidity post PPH is thus virtually unknown.

From the 2210 papers identified in this review, only six reported on outcomes following PPH. Of these, we were unable to pool and meta-analyse most data because of differing definitions of PPH, differing timing of measurement or differing outcomes measured. Those that could be pooled demonstrated a 0.33 % rate of acute renal failure and a 1.74 % rate of coagulopathy. The re-admission rate to hospital following a PPH between 1 and 3 months postpartum was 3.6 %, an appreciable indication of underlying physical problems. Single studies included in this review showed other types of morbidity to be common; for example, depression (13 %) and a health status at 6 months postpartum, as measured by SF36, as ‘much worse than 1 year ago’ (6 %) [27].

Few studies of emotional sequelae of PPH within the six month postpartum period were found. Some work has shown that 68 % of 68 women surviving severe PPH had negative memories of the event (such as fear of dying (35 %)) when interviewed 4 to 17 years later) [5]. Sixty percent of the 15 women who went on to have another pregnancy suffered severe anxiety throughout, indicating the tenacity of such feelings. If emotional morbidity from PPH could be detected in the early postpartum months, and treated, it is possible that such long-term psychological disability could be averted. The first step, however, is to measure the prevalence and severity of such morbidity and an observational study is in progress in France that may provide more information in this area [37].

Secondary PPH is poorly defined, yet it seems illogical to define it as a blood loss of 500 mls, the amount used to define primary PPH, because this is considered to be in excess of what is considered a physiologically normal blood loss during the third stage of labour. We suggest that the definition should more correctly be: “any blood loss from the gentital tract in excess of normal lochia at any time period after 24 h post birth to 6 weeks postpartum.”

It is clear that more research is needed, to ascertain the amount and degree of both physical and emotional postpartum morbidity following PPH. Before that, though, it is important to develop a core outcomes set [38] for measuring maternal health outcomes postpartum, perhaps as a subset of the maternity core outcome set developed by Devane et al. [39].

### Strengths and limitations of this review

The findings from this review are based on a comprehensive systematic literature search of seven electronic databases, and rigorous critical appraisal of included studies. Two studies with large sample sizes [28, 30] influenced the overall results. Unfortunately there was high heterogeneity between all studies, which is a major limitation. We tried to be as inclusive as possible, but we were unable to pool and meta-analyse most of the findings, thus limiting the review’s potential to add new knowledge to clinical practice. Despite this limitation, the findings demonstrate that the prevalence of physical and emotional health problems experienced by women post PPH remain largely unknown, indicating the need for further research in this area.

## Conclusion

This review demonstrates that there are few studies examining the prevalence of maternal physical and health problems post PPH, and those that were found tended to be either small and/or focussed on a limited number of morbidities. PPH, estimated to affect as many as 8–11 million women annually, is likely to impact negatively on women’s health, and it is conceivable that existing health problems experienced may be exacerbated. As these health problems remain virtually unknown, however, many women may be suffering sequelae from PPH during the postpartum period in the community, without proper diagnosis or treatment. Further research is required, but because of the variations in definitions used, and outcomes reported, studies will require a clear definition of PPH, and valid and reliable measuring instruments, based on a core outcome set, to ascertain the timing, prevalence and severity of health problems.

## Additional file

Additional file 1: Search strings for databases. (DOCX 24.7 kb)

## Abbreviations

CS: Caesarean section
DIC: Disseminated Intravascular Coagulation
NOS: Newcastle Ottawa Scale
PPH: Postpartum haemorrhage
