# Supplementary material for: The prevalence of women’s emotional and physical health problems following a postpartum haemorrhage: a systematic review
Source: BMC Pregnancy Childbirth. 2016 Sep 5;16(1):261. doi: 10.1186/s12884-016-1054-1 (PMC5011962; doi:10.1186/s12884-016-1054-1)
Supplement: Additional file 1: — Search strings for databases. (DOCX 24.7 kb) [file 12884_2016_1054_MOESM1_ESM.docx]

**The Prevalence of Women’s Emotional and Physical Health Problems following a Postpartum Haemorrhage: A Systematic Review**

## Search Strings

**Search String for CINAHL and PsycINFO**

| **CINAHL** |  |
| --- | --- |
| TI postnatal OR AB postnatal OR TI post natal OR AB post natal OR TI post-natal OR AB post-natal OR TI postpartum OR AB postpartum OR TI post partum OR AB post partum OR TI post-partum OR AB post-partum OR TI "postnatal period" OR AB "postnatal period" OR TI "post natal period" OR AB "post natal period" OR TI "post-natal period" OR AB "post-natal period" OR TI "postpartum period" OR AB "postpartum period" OR TI "post partum period" OR AB "post partum period" OR TI "post-partum period" OR AB "post-partum period" OR TI puerperium OR AB Puerperium OR TI peripartum OR AB peripartum OR TI peri partum OR AB peri partum OR TI peri-partum OR AB peri-partum OR TI "peripartum period" OR AB "peripartum period" OR TI "peri partum period" OR AB "peri partum period" OR TI "peri-partum period" OR AB "peri-partum period" OR TI peripartum period OR AB peripartum period | 13,420 |
| TI PPH OR AB PPH OR TI postpartum haemorrhage OR AB postpartum haemorrhage OR TI post partum haemorrhage OR AB post partum haemorrhage OR TI post-partum haemorrhage OR AB post-partum haemorrhage OR TI postpartum hemorrhage OR AB postpartum hemorrhage OR TI post partum hemorrhage OR AB post partum hemorrhage OR TI post-partum hemorrhage OR AB post-partum hemorrhage OR TI obstetric haemorrhage OR AB obstetric haemorrhage OR TI obstetric hemorrhage OR AB obstetric hemorrhage OR TI major obstetric haemorrhage OR AB major obstetric haemorrhage OR TI major obstetric hemorrhage OR AB major obstetric hemorrhage OR TI massive haemorrhage OR AB massive haemorrhage OR TI massive hemorrhage OR AB massive hemorrhage OR TI massive obstetric haemorrhage OR AB massive obstetric haemorrhage OR TI massive obstetric hemorrhage OR AB massive obstetric hemorrhage OR TI severe haemorrhage OR AB severe haemorrhage OR TI severe hemorrhage OR AB severe hemorrhage OR TI severe obstetric haemorrhage OR AB severe obstetric haemorrhage OR TI severe obstetric hemorrhage OR AB severe obstetric hemorrhage OR TI severe blood loss OR AB severe blood loss | 1,596 |
| TI women’s health OR AB women’s health OR TI womens health OR AB womens health OR TI woman’s health OR AB woman’s health OR TI womans health OR AB womans health OR TI health outcome OR AB health outcome OR TI health outcomes OR AB health outcomes OR TI adverse outcome OR AB adverse outcome OR TI adverse outcomes OR AB adverse outcomes OR TI pregnancy outcome OR AB pregnancy outcome OR TI pregnancy outcomes OR AB pregnancy outcomes OR TI puerperal disorder OR AB puerperal disorder OR TI puerperal disorders OR AB puerperal disorders OR TI maternal morbidity OR AB maternal morbidity OR TI maternal morbidities OR AB maternal morbidities OR TI mental health OR AB mental health OR TI mental disorder OR AB mental disorder OR TI mental disorders OR AB mental disorders OR TI mental disease OR AB mental disease OR TI mental diseases OR AB mental diseases OR TI emotional wellbeing OR AB emotional wellbeing OR TI emotional well being OR AB emotional well being OR TI emotional well-being OR AB emotional well-being OR TI emotional health OR AB emotional health OR TI postnatal depression OR AB postnatal depression OR TI post natal depression OR AB post natal depression OR TI post-natal depression OR AB post-natal depression OR TI postpartum depression OR AB postpartum depression OR TI post partum depression OR AB post partum depression OR TI post-partum depression OR AB post-partum depression OR TI postnatal distress OR AB postnatal distress OR TI post natal distress OR AB post natal distress OR TI post-natal distress OR AB post-natal distress OR TI postpartum distress OR AB postpartum distress OR TI post partum distress OR AB post partum distress OR TI post-partum distress OR AB post-partum distress OR TI postnatal stress OR AB postnatal stress OR TI post natal stress OR AB post natal stress OR TI post-natal stress OR AB post-natal stress OR TI perinatal mental health OR AB perinatal mental health OR TI perinatal mental health problems OR AB perinatal mental health problems OR TI peri natal mental health problems OR AB peri natal mental health problems Or TI perinatal mental health problem OR AB perinatal mental health problem OR TI peri natal mental health problem OR AB peri natal mental health problem OR TI postnatal anxiety OR AB postnatal anxiety OR TI post natal anxiety OR AB post natal anxiety OR TI post-natal anxiety OR AB post-natal anxiety OR TI postpartum anxiety OR AB postpartum anxiety OR TI post partum anxiety OR AB post partum anxiety OR TI post-partum anxiety OR AB post-partum anxiety OR TI postnatal anxieties OR AB postnatal anxieties OR TI post natal anxieties OR AB post natal anxieties OR TI post-natal anxieties OR AB post-natal anxieties OR TI postpartum anxieties OR AB postpartum anxieties OR TI post partum anxieties OR AB post partum anxieties OR TI post-partum anxieties OR AB post-partum anxieties OR TI postnatal psychosis OR AB postnatal psychosis OR TI post natal psychosis OR AB post natal psychosis OR TI post-natal psychosis OR AB post-natal psychosis OR TI postpartum psychosis OR AB postpartum psychosis OR TI post partum psychosis OR AB post partum psychosis OR TI post-partum psychosis OR AB post-partum psychosis OR TI physical health OR AB physical health OR TI physical health problem OR AB physical health problem OR TI physical health problems OR AB physical health problems OR TI health problem OR AB health problem OR TI health problems OR AB health problems | 100,412 |
| Combined with AND | 162 |
| **PsycINFO** |  |
| Postnatal period (same string as above) | 22,407 |
| PPH (same string as above) | 289 |
| Women’s emotional and physical health problems (same string as above) | 227,052 |
| Combined with AND | 27 |

**Search string for Web of Science**

| **Web of Science (all databases) formally known as Web of Knowledge 1990 to present** | |
| --- | --- |
| TI=(postnatal OR "post natal" OR "postnatal period" OR "post natal period" OR postpartum OR “postpartum period" OR "post partum period" OR Puerperium OR peripartum OR “peri partum” OR “peripartum period” OR “peri partum period”) OR Ts=(postnatal OR "post natal" OR "postnatal period" OR "post natal period" OR postpartum OR “postpartum period" OR "post partum period" OR Puerperium OR peripartum OR “peri partum” OR “peripartum period” OR “peri partum period”) | 579,146 |
| TI=(“pph” OR “postpartum haemorrhage” OR “postpartum hemorrhage” OR “post partum haemorrhage” OR “post partum hemorrhage” OR “obstetric haemorrhage” OR “obstetric hemorrhage” OR “major obstetric haemorrhage” OR “major obstetric hemorrhage” OR “massive obstetric haemorrhage” OR “massive obstetric hemorrhage” OR “massive haemorrhage” OR “massive hemorrhage” OR “severe haemorrhage” OR “severe hemorrhage” OR “severe obstetric haemorrhage” OR “severe obstetric hemorrhage” OR “severe blood loss ”) OR Ts=(“pph” OR “postpartum haemorrhage” OR “postpartum hemorrhage” OR “post partum haemorrhage” OR “post partum hemorrhage” OR “obstetric haemorrhage” OR “obstetric hemorrhage” OR “major obstetric haemorrhage” OR “major obstetric hemorrhage” OR “massive obstetric haemorrhage” OR “massive obstetric hemorrhage” OR “massive haemorrhage” OR “massive hemorrhage” OR “severe haemorrhage” OR “severe hemorrhage” OR “severe obstetric haemorrhage” OR “severe obstetric hemorrhage” OR “severe blood loss ”) | 53,238 |
| Ti=(“women’s health” OR “womens health” OR “woman’s health” OR “womans health” OR “health outcome” OR “health outcomes” OR “adverse outcome” OR “adverse outcomes” OR “pregnancy outcome” OR “pregnancy outcomes” OR “puerperal disorder” OR “puerperal disorders” OR “maternal morbidity” OR “maternal morbidities” OR “mental health” OR “mental disorder” OR “mental disorders” OR “mental disease” OR “mental diseases” OR “emotional wellbeing” OR “emotional well being” OR “emotional well-being” OR “emotional health” OR “postnatal depression” OR “post natal depression” OR “post-natal depression” OR “postpartum depression” OR “post partum depression” OR “post-partum depression” OR “postnatal distress” OR “post natal distress” OR “post-natal distress” OR “postpartum distress” OR “post partum distress” OR “post-partum distress” OR “postnatal stress” OR “post natal stress” OR “post-natal stress” OR “perinatal mental health” OR “perinatal mental health problems” OR “peri natal mental health problems” OR “perinatal mental health problem” OR “peri natal mental health problem” OR “postnatal anxiety” OR “post natal anxiety” OR “post-natal anxiety” OR “postpartum anxiety” OR “post partum anxiety” OR “post-partum anxiety” OR “postnatal anxieties” OR “post natal anxieties” OR “post-natal anxieties” OR “postpartum anxieties” OR “post partum anxieties” OR “post-partum anxieties” OR “postnatal psychosis” OR “post natal psychosis” OR “post-natal psychosis” OR “postpartum psychosis” OR “post partum psychosis” OR “post-partum psychosis” OR “physical health” OR “physical health problem” OR “physical health problems” OR “health problem” OR “health problems”) OR Ts=(“women’s health” OR “womens health” OR “woman’s health” OR “womans health” OR “health outcome” OR “health outcomes” OR “adverse outcome” OR “adverse outcomes” OR “pregnancy outcome” OR “pregnancy outcomes” OR “puerperal disorder” OR “puerperal disorders” OR “maternal morbidity” OR “maternal morbidities” OR “mental health” OR “mental disorder” OR “mental disorders” OR “mental disease” OR “mental diseases” OR “emotional wellbeing” OR “emotional well being” OR “emotional well-being” OR “emotional health” OR “postnatal depression” OR “post natal depression” OR “post-natal depression” OR “postpartum depression” OR “post partum depression” OR “post-partum depression” OR “postnatal distress” OR “post natal distress” OR “post-natal distress” OR “postpartum distress” OR “post partum distress” OR “post-partum distress” OR “postnatal stress” OR “post natal stress” OR “post-natal stress” OR “perinatal mental health” OR “perinatal mental health problems” OR “peri natal mental health problems” OR “perinatal mental health problem” OR “peri natal mental health problem” OR “postnatal anxiety” OR “post natal anxiety” OR “post-natal anxiety” OR “postpartum anxiety” OR “post partum anxiety” OR “post-partum anxiety” OR “postnatal anxieties” OR “post natal anxieties” OR “post-natal anxieties” OR “postpartum anxieties” OR “post partum anxieties” OR “post-partum anxieties” OR “postnatal psychosis” OR “post natal psychosis” OR “post-natal psychosis” OR “postpartum psychosis” OR “post partum psychosis” OR “post-partum psychosis” OR “physical health” OR “physical health problem” OR “physical health problems” OR “health problem” OR “health problems”) | 1,873,210 |
| Combined with AND | 1,747 |

**Search String for EMBASE**

| postnatal OR ‘post natal’ OR ‘postnatal period’ OR ‘post natal period’ OR postpartum OR ‘post partum’ OR ‘postpartum period’ OR ‘post partum period’ OR Puerperium OR peripartum OR ‘peri partum’ OR ‘peripartum period’ OR ‘peri partum period’) OR Ts=(postnatal OR ‘post natal’ OR ‘postnatal period’ OR ‘post natal period’ OR postpartum OR ‘post partum’ OR ‘postpartum period’ OR ‘post partum period’ OR Puerperium OR peripartum OR ‘peri partum’ OR ‘peripartum period’ OR ‘peri partum period’ | 93,737 |
| --- | --- |
| ‘PPH’ OR ‘postpartum haemorrhage’ OR ‘post partum haemorrhage’ OR ‘postpartum haemorrhage- OR ‘post partum hemorrhage’ OR ‘obstetric haemorrhage’ OR ‘obstetric hemorrhage’ OR ‘major obstetric haemorrhage’ OR ‘major obstetric hemorrhage’ OR ‘massive haemorrhage’ OR ‘massive hemorrhage’ OR ‘massive obstetric haemorrhage’ OR ‘massive obstetric hemorrhage’ OR ‘severe haemorrhage’ OR ‘severe hemorrhage’ OR ‘severe obstetric haemorrhage’ OR ‘severe obstetric hemorrhage’ OR ‘severe blood loss’ | 9,335 |
| ‘women health’ OR ‘womens health’ OR ‘woman health’ OR ‘womans health’ OR ‘health outcome’ OR ‘health outcomes’ OR ‘adverse outcome’ OR ‘adverse outcomes’ OR ‘pregnancy outcome’ OR ‘pregnancy outcomes’ OR ‘puerperal disorder’ OR ‘puerperal disorders’ OR ‘maternal morbidity’ OR ‘maternal morbidities’ OR ‘mental health’ OR ‘mental disorder’ OR ‘mental disorders’ OR ‘mental disease’ OR ‘mental diseases’ OR ‘emotional wellbeing’ OR ‘emotional well being’ OR ‘emotional well-being’ OR ‘emotional health’ OR ‘postnatal depression’ OR ‘post natal depression’ OR ‘post-natal depression’ OR ‘postpartum depression’ OR ‘post partum depression’ OR ‘post-partum depression’ OR ‘postnatal distress’ OR ‘post natal distress’ OR ‘post-natal distress’ OR ‘postpartum distress’ OR ‘post partum distress’ OR ‘post-partum distress’ OR ‘postnatal stress’ OR ‘post natal stress’ OR ‘post-natal stress’ OR ‘perinatal mental health’ OR ‘perinatal mental health problems’ OR ‘peri natal mental health problems’ OR ‘perinatal mental health problem’ OR ‘peri natal mental health problem’ OR ‘postnatal anxiety’ OR ‘post natal anxiety’ OR ‘post-natal anxiety’ OR ‘postpartum anxiety’ OR ‘post partum anxiety’ OR ‘post-partum anxiety’ OR ‘postnatal anxieties’ OR ‘post natal anxieties’ OR ‘post-natal anxieties’ OR ‘postpartum anxieties’ OR ‘post partum anxieties’ OR ‘post-partum anxieties’ OR ‘postnatal psychosis’ OR ‘post natal psychosis’ OR ‘post-natal psychosis’ OR ‘postpartum psychosis’ OR ‘post partum psychosis’ OR ‘post-partum psychosis’ OR ‘physical health’ OR ‘physical health problem’ OR ‘physical health problems’ OR ‘health problem’ OR ‘health problems’ | 638,872 |
| Combined with AND | 1,003 |

**Search String for Maternity and Infant Care**

| (postnatal or post natal or postnatal period or post natal period or postpartum or post partum or postpartum period or post partum period or puerperium or peripartum or peri partum or peripartum period or peri partum period).ab,ti. | 20,541 |
| --- | --- |
| (pph or postpartum haemorrhage or postpartum hemorrhage or post partum haemorrhage or post partum hemorrhage or obstetric haemorrhage or obstetric hemorrhage or major obstetric haemorrhage or major obstetric hemorrhage or massive haemorrhage or massive hemorrhage or massive obstetric haemorrhage or massive obstetric hemorrhage or severe haemorrhage or severe hemorrhage or severe obstetric haemorrhage or severe obstetric hemorrhage or severe blood loss).ab,ti. | 2,146 |
| (womens health or womens health or womans health or womans health or health outcome or health outcomes or adverse outcome or adverse outcomes or pregnancy outcome or pregnancy outcomes or puerperal disorder or puerperal disorders or maternal morbidity or maternal morbidities or mental health or mental disorder or mental disorders or mental disease or mental diseases or emotional wellbeing or emotional well being or emotional health or postnatal depression or post natal depression or postpartum depression or post partum depression or postnatal distress or post natal distress or postpartum distress or post partum distress or postnatal stresss or post natal stress or perinatal mental health or perinatal mental health problems or peri natal mental health problems or perinatal mental health problem or peri natal mental health problem or postnatal anxiety or post natal anxiety or postpartum anxiety or post partum anxiety or postnatal anxieties or post natal anxieties or postpartum anxieties or post partum anxieties or postnatal psychosis or post natal psychosis or postpartum psychosis or post partum psychosis or physical health or physical health problem or physical health problems or health problem or health problems).ab,ti. | 15,034 |
| Combined with AND | 352 |

**Search String for COCHRANE LIBRARY**

| postnatal or post natal or postnatal period or post natal period or postpartum or post partum or postpartum period or post partum period or puerperium or peripartum or peri partum or peripartum period or peri partum period:ti (Word variations have been searched)  postnatal or post natal or postnatal period or post natal period or postpartum or post partum or postpartum period or post partum period or puerperium or peripartum or peri partum or peripartum period or peri partum period:ab (Word variations have been searched) | 5,320 |
| --- | --- |
| pph or postpartum haemorrhage or postpartum hemorrhage or post partum haemorrhage or post partum hemorrhage or obstetric haemorrhage or obstetric hemorrhage or major obstetric haemorrhage or major obstetric hemorrhage or massive haemorrhage or massive hemorrhage or massive obstetric haemorrhage or massive obstetric hemorrhage or severe haemorrhage or severe hemorrhage or severe obstetric haemorrhage or severe obstetric hemorrhage or severe blood loss:ti (Word variations have been searched)  pph or postpartum haemorrhage or postpartum hemorrhage or post partum haemorrhage or post partum hemorrhage or obstetric haemorrhage or obstetric hemorrhage or major obstetric haemorrhage or major obstetric hemorrhage or massive haemorrhage or massive hemorrhage or massive obstetric haemorrhage or massive obstetric hemorrhage or severe haemorrhage or severe hemorrhage or severe obstetric haemorrhage or severe obstetric hemorrhage or severe blood loss:ab (Word variations have been searched) | 2,021 |
| womens health or womens health or womans health or womans health or health outcome or health outcomes or adverse outcome or adverse outcomes or pregnancy outcome or pregnancy outcomes or puerperal disorder or puerperal disorders or maternal morbidity or maternal morbidities or mental health or mental disorder or mental disorders or mental disease or mental diseases or emotional wellbeing or emotional well being or emotional health or postnatal depression or post natal depression or postpartum depression or post partum depression or postnatal distress or post natal distress or postpartum distress or post partum distress or postnatal stresss or post natal stress or perinatal mental health or perinatal mental health problems or peri natal mental health problems or perinatal mental health problem or peri natal mental health problem or postnatal anxiety or post natal anxiety or postpartum anxiety or post partum anxiety or postnatal anxieties or post natal anxieties or postpartum anxieties or post partum anxieties or postnatal psychosis or post natal psychosis or postpartum psychosis or post partum psychosis or physical health or physical health problem or physical health problems or health problem or health problems:ti (Word variations have been searched)  womens health or womens health or womans health or womans health or health outcome or health outcomes or adverse outcome or adverse outcomes or pregnancy outcome or pregnancy outcomes or puerperal disorder or puerperal disorders or maternal morbidity or maternal morbidities or mental health or mental disorder or mental disorders or mental disease or mental diseases or emotional wellbeing or emotional well being or emotional health or postnatal depression or post natal depression or postpartum depression or post partum depression or postnatal distress or post natal distress or postpartum distress or post partum distress or postnatal stresss or post natal stress or perinatal mental health or perinatal mental health problems or peri natal mental health problems or perinatal mental health problem or peri natal mental health problem or postnatal anxiety or post natal anxiety or postpartum anxiety or post partum anxiety or postnatal anxieties or post natal anxieties or postpartum anxieties or post partum anxieties or postnatal psychosis or post natal psychosis or postpartum psychosis or post partum psychosis or physical health or physical health problem or physical health problems or health problem or health problems:ab (Word variations have been searched) | 53,514 |
| Combined with AND | 206 |

**Search String for PUBMED**

| (("peri partum period"[title/abstract] OR "peripartum period"[title/abstract] OR "peri partum"[title/abstract] OR peripartum[title/abstract] OR puerperium[title/abstract] OR "post partum period"[Title/abstract] OR "postpartum period"[Title/abstract] OR "post partum"[Title/Abstract] OR "postpartum"[Title/Abstract] OR "post natal period"[Title/Abstract] OR "postnatal period"[Title/Abstract] OR "post natal"[Title/Abstract] OR postnatal[Title/Abstract]) AND (PPH[Title/Abstract] OR "postpartum haemorrhage"[Title/Abstract] OR "post partum haemorrhage"[Title/Abstract] OR "postpartum hemorrhage"[Title/Abstract] OR "post partum hemorrhage"[Title/Abstract] OR "obstetric haemorrhage"[Title/Abstract] OR "obstetric hemorrhage"[Title/Abstract] OR "major obstetric haemorrhage"[Title/Abstract] OR "major obstetric hemorrhage"[Title/Abstract] OR "massive obstetric haemorrhage"[Title/Abstract] OR "massive obstetric hemorrhage"[Title/Abstract] OR "massive haemorrhage"[Title/Abstract] OR "massive hemorrhage"[Title/Abstract] OR "severe haemorrhage"[Title/Abstract] OR "severe hemorrhage"[Title/Abstract] OR "severe obstetric haemorrhage"[Title/Abstract] OR "severe obstetric hemorrhage"[Title/Abstract] OR "severe blood loss"[Title/Abstract])) AND ("women's health"[Title/Abstract] OR "womens health"[Title/Abstract] OR "woman's health"[Title/Abstract] OR "health outcome"[Title/Abstract] OR "health outcomes"[Title/Abstract] OR "adverse outcome"[Title/Abstract] OR "adverse outcomes"[Title/Abstract] OR "pregnancy outcome"[Title/Abstract] OR "pregnancy outcomes"[Title/Abstract] OR "puerperal disorder"[Title/Abstract] OR "puerperal disorders"[Title/Abstract] OR "maternal morbidity"[Title/Abstract] OR "maternal morbidities"[Title/Abstract] OR "mental health"[Title/Abstract] OR "mental disorder"[Title/Abstract] OR "mental disorders"[Title/Abstract] OR "mental disease"[Title/Abstract] OR "mental diseases"[Title/Abstract] OR "emotional wellbeing"[Title/Abstract] OR "emotional well being"[Title/Abstract] OR "emotional well-being"[Title/Abstract] OR "emotional health"[Title/Abstract] OR "postnatal depression"[Title/Abstract] OR "post natal depression"[Title/Abstract] OR "post-natal depression"[Title/Abstract] OR "postpartum depression"[Title/Abstract] OR "post partum depression"[Title/Abstract] OR "post-partum depression"[Title/Abstract] OR "postnatal distress"[Title/Abstract] OR "postpartum distress"[Title/Abstract] OR "post partum distress"[Title/Abstract] OR "post-partum distress"[Title/Abstract] OR "postnatal stress"[Title/Abstract] OR "post natal stress"[Title/Abstract] OR "post-natal stress"[Title/Abstract] OR "perinatal mental health"[Title/Abstract] OR "perinatal mental health problems"[Title/Abstract] OR "postnatal anxiety"[Title/Abstract] OR "post natal anxiety"[Title/Abstract] OR "post-natal anxiety"[Title/Abstract] OR "postpartum anxiety"[Title/Abstract] OR "post partum anxiety"[Title/Abstract] OR "post-partum anxiety"[Title/Abstract] OR "postnatal psychosis"[Title/Abstract] OR "postpartum psychosis"[Title/Abstract] OR "post partum psychosis"[Title/Abstract] OR "post-partum psychosis"[Title/Abstract] OR "physical health"[Title/Abstract] OR "physical health problem"[Title/Abstract] OR "physical health problems"[Title/Abstract] OR "health problem"[Title/Abstract] OR "health problems"[Title/Abstract]  **Following phrases not found**  "womans health"[Title/Abstract]  "post natal distress"[Title/Abstract]  "post-natal distress"[Title/Abstract]  "peri natal mental health problems"[Title/Abstract]  "perinatal mental health problem"[Title/Abstract]  "peri natal mental health problem"[Title/Abstract]  "postnatal anxieties"[Title/Abstract]  "post natal anxieties"[Title/Abstract]  "post-natal anxieties"[Title/Abstract]  "postpartum anxieties"[Title/Abstract]  "post partum anxieties"[Title/Abstract]  "post-partum anxieties"[Title/Abstract]  "post natal psychosis"[Title/Abstract]  "post-natal psychosis"[Title/Abstract] | 613 |
| --- | --- |

**Search String for Social Science Index**

| TI postnatal OR AB postnatal OR TI post natal OR AB post natal OR TI post-natal OR AB post-natal OR TI postpartum OR AB postpartum OR TI post partum OR AB post partum OR TI post-partum OR AB post-partum OR TI "postnatal period" OR AB "postnatal period" OR TI "post natal period" OR AB "post natal period" OR TI "post-natal period" OR AB "post-natal period" OR TI "postpartum period" OR AB "postpartum period" OR TI "post partum period" OR AB "post partum period" OR TI "post-partum period" OR AB "post-partum period" OR TI puerperium OR AB Puerperium OR TI peripartum OR AB peripartum OR TI peri partum OR AB peri partum OR TI peri-partum OR AB peri-partum OR TI "peripartum period" OR AB "peripartum period" OR TI "peri partum period" OR AB "peri partum period" OR TI "peri-partum period" OR AB "peri-partum period" OR TI peripartum period OR AB peripartum period | 1,210 |
| --- | --- |
| TI PPH OR AB PPH OR TI postpartum haemorrhage OR AB postpartum haemorrhage OR TI post partum haemorrhage OR AB post partum haemorrhage OR TI post-partum haemorrhage OR AB post-partum haemorrhage OR TI postpartum hemorrhage OR AB postpartum hemorrhage OR TI post partum hemorrhage OR AB post partum hemorrhage OR TI post-partum hemorrhage OR AB post-partum hemorrhage OR TI obstetric haemorrhage OR AB obstetric haemorrhage OR TI obstetric hemorrhage OR AB obstetric hemorrhage OR TI major obstetric haemorrhage OR AB major obstetric haemorrhage OR TI major obstetric hemorrhage OR AB major obstetric hemorrhage OR TI massive haemorrhage OR AB massive haemorrhage OR TI massive hemorrhage OR AB massive hemorrhage OR TI massive obstetric haemorrhage OR AB massive obstetric haemorrhage OR TI massive obstetric hemorrhage OR AB massive obstetric hemorrhage OR TI severe haemorrhage OR AB severe haemorrhage OR TI severe hemorrhage OR AB severe hemorrhage OR TI severe obstetric haemorrhage OR AB severe obstetric haemorrhage OR TI severe obstetric hemorrhage OR AB severe obstetric hemorrhage OR TI severe blood loss OR AB severe blood loss | 22 |
| TI women’s health OR AB women’s health OR TI womens health OR AB womens health OR TI woman’s health OR AB woman’s health OR TI womans health OR AB womans health OR TI health outcome OR AB health outcome OR TI health outcomes OR AB health outcomes OR TI adverse outcome OR AB adverse outcome OR TI adverse outcomes OR AB adverse outcomes OR TI pregnancy outcome OR AB pregnancy outcome OR TI pregnancy outcomes OR AB pregnancy outcomes OR TI puerperal disorder OR AB puerperal disorder OR TI puerperal disorders OR AB puerperal disorders OR TI maternal morbidity OR AB maternal morbidity OR TI maternal morbidities OR AB maternal morbidities OR TI mental health OR AB mental health OR TI mental disorder OR AB mental disorder OR TI mental disorders OR AB mental disorders OR TI mental disease OR AB mental disease OR TI mental diseases OR AB mental diseases OR TI emotional wellbeing OR AB emotional wellbeing OR TI emotional well being OR AB emotional well being OR TI emotional well-being OR AB emotional well-being OR TI emotional health OR AB emotional health OR TI postnatal depression OR AB postnatal depression OR TI post natal depression OR AB post natal depression OR TI post-natal depression OR AB post-natal depression OR TI postpartum depression OR AB postpartum depression OR TI post partum depression OR AB post partum depression OR TI post-partum depression OR AB post-partum depression OR TI postnatal distress OR AB postnatal distress OR TI post natal distress OR AB post natal distress OR TI post-natal distress OR AB post-natal distress OR TI postpartum distress OR AB postpartum distress OR TI post partum distress OR AB post partum distress OR TI post-partum distress OR AB post-partum distress OR TI postnatal stress OR AB postnatal stress OR TI post natal stress OR AB post natal stress OR TI post-natal stress OR AB post-natal stress OR TI perinatal mental health OR AB perinatal mental health OR TI perinatal mental health problems OR AB perinatal mental health problems OR TI peri natal mental health problems OR AB peri natal mental health problems Or TI perinatal mental health problem OR AB perinatal mental health problem OR TI peri natal mental health problem OR AB peri natal mental health problem OR TI postnatal anxiety OR AB postnatal anxiety OR TI post natal anxiety OR AB post natal anxiety OR TI post-natal anxiety OR AB post-natal anxiety OR TI postpartum anxiety OR AB postpartum anxiety OR TI post partum anxiety OR AB post partum anxiety OR TI post-partum anxiety OR AB post-partum anxiety OR TI postnatal anxieties OR AB postnatal anxieties OR TI post natal anxieties OR AB post natal anxieties OR TI post-natal anxieties OR AB post-natal anxieties OR TI postpartum anxieties OR AB postpartum anxieties OR TI post partum anxieties OR AB post partum anxieties OR TI post-partum anxieties OR AB post-partum anxieties OR TI postnatal psychosis OR AB postnatal psychosis OR TI post natal psychosis OR AB post natal psychosis OR TI post-natal psychosis OR AB post-natal psychosis OR TI postpartum psychosis OR AB postpartum psychosis OR TI post partum psychosis OR AB post partum psychosis OR TI post-partum psychosis OR AB post-partum psychosis OR TI physical health OR AB physical health OR TI physical health problem OR AB physical health problem OR TI physical health problems OR AB physical health problems OR TI health problem OR AB health problem OR TI health problems OR AB health problems | 26,657 |
| Combined with AND | 2 |

**Results of Search 17/04/2015**

| **Data base** | |
| --- | --- |
| CINAHL | 162 |
| PsycINFO | 27 |
| Web of Science | 1747 |
| EMBASE | 1,003 |
| MEDLINE (not searched) |  |
| Maternity and Infant Care | 352 |
| COCHRANE LIBRARY | 206 |
| PubMed | 613 |
| Social Science Index | 2 |
| Total | 4112 |
